# Unexpected CA 19–9 Elevation in a Patient With a Splenic Epithelial Cyst: A Diagnostic Challenge

**DOI:** 10.7759/cureus.89697

**Published:** 2025-08-09

**Authors:** Mohammed A Alsuhaimi, Mohmmed A AlHewishel, May S AlKhaldi, Abdurahman S AlSumaihi, Khaled S Albahout

**Affiliations:** 1 Surgery, Dammam Medical Complex, Dammam, SAU; 2 General Surgery, Dammam Medical Complex, Dammam, SAU; 3 General Surgery, King Fahad University Hospital, Khobar, SAU

**Keywords:** benign splenic lesion, ca 19-9, laparoscopic splenectomy, non-malignant ca 19-9 elevation, splenic epithelial cyst

## Abstract

Splenic epithelial cysts are rare, benign lesions often discovered incidentally. Although typically asymptomatic, they can sometimes present with elevated tumor markers, such as CA 19-9, raising concerns for malignancy. We report the case of a 44-year-old woman who was found to have elevated liver enzymes and CA 19-9 levels. Imaging revealed a unilocular splenic cyst. Laparoscopic splenectomy was performed, and histopathology confirmed a benign epithelial cyst. Postoperatively, CA 19-9 levels declined significantly. This case highlights the importance of considering benign etiologies in patients with elevated tumor markers and emphasizes the role of imaging and surgical pathology in establishing a diagnosis.

## Introduction

Splenic cysts are rare, with an incidence of approximately 0.07% based on autopsy studies, and they comprise a heterogeneous group of lesions classified as parasitic and non-parasitic [1,2]. Non-parasitic splenic cysts are further divided into true (epithelial-lined) cysts and pseudocysts, the latter commonly resulting from trauma or infarction [1,3]. Among true cysts, splenic epithelial cysts are the most frequently reported subtype, although they remain clinically uncommon.

Most splenic epithelial cysts are discovered incidentally during imaging for unrelated conditions. They are typically asymptomatic, but large cysts may cause abdominal discomfort, early satiety, or a palpable mass [2]. In some cases, they may rupture, bleed, or become infected, requiring surgical intervention [3].

A challenging and noteworthy aspect of splenic epithelial cysts is their association with elevated serum tumor markers, particularly carbohydrate antigen 19-9 (CA 19-9), a glycoprotein typically associated with malignancies of the pancreas, biliary tract, and gastrointestinal system [4,5]. Although rare, splenic epithelial cysts account for approximately 10% of non-parasitic splenic cysts, and non-parasitic cysts in general make up less than 1% of all splenic lesions [1,2]. The presence of elevated CA 19-9 in a patient with a splenic lesion often raises concern for malignancy, which can lead to unnecessary investigations or overtreatment.

Recent studies suggest that this elevation may originate from the secretion of CA 19-9 by the epithelial lining of the cyst, especially when intracystic pressure increases due to growth or partial rupture [6,7]. Recognition of this benign mechanism is critical in avoiding misdiagnosis. We present a case of a large splenic epithelial cyst in an asymptomatic woman with elevated CA 19-9, successfully managed by laparoscopic splenectomy, with complete resolution of the biochemical abnormality.

## Case presentation

A 44-year-old female patient with a background of type 2 diabetes mellitus was assessed for increased liver enzyme levels. During this evaluation, an exceptionally high CA 19-9 level (627.65 U/mL) was detected. The patient was asymptomatic and denied any history of trauma, malignancy, or previous surgical procedures. Physical examination indicated a fullness in the left upper quadrant without accompanying discomfort, and the remainder of the systemic examination was normal. Laboratory tests showed normal levels of carcinoembryonic antigen (CEA) at 2.17 µg/L (Table 1). Liver function tests, including AST, ALT, ALP, and total bilirubin, were all within normal reference ranges, indicating no hepatobiliary involvement.

**Table 1:** Summary of laboratory investigations at initial presentation

Test	Patient result	Reference range	Units
CA 19-9	627.65	0–37	U/mL
CEA	2.17	0–5	µg/L
ALT	82	7–56	U/L
AST	70	5–40	U/L
ALP	112	44–147	U/L
Total bilirubin	1.2	0.1–1.2	mg/dL
Creatinine	0.8	0.6–1.2	mg/dL
WBC	6.3	4.0–11.0	x10⁹/L
Hemoglobin	13.4	12.0–16.0	g/dL

CT findings

**Figure 1:**
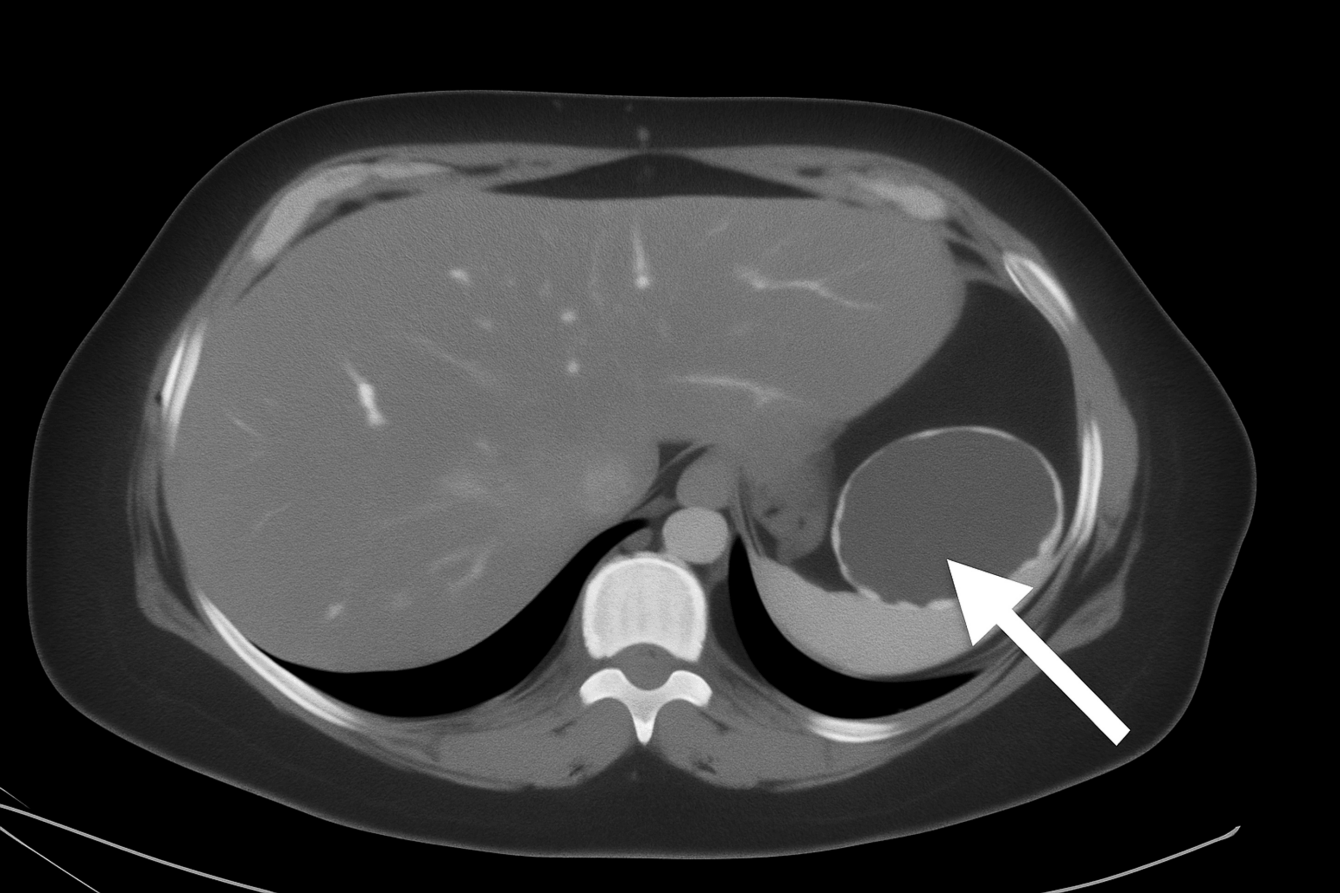
Axial contrast-enhanced CT scan demonstrating a splenic epithelial cyst A well-defined, unilocular cystic lesion is seen in the upper pole of the spleen (white arrow), measuring approximately 6–7 cm, with no internal septations or solid components. Findings are consistent with a benign epithelial cyst.

**Figure 2:**
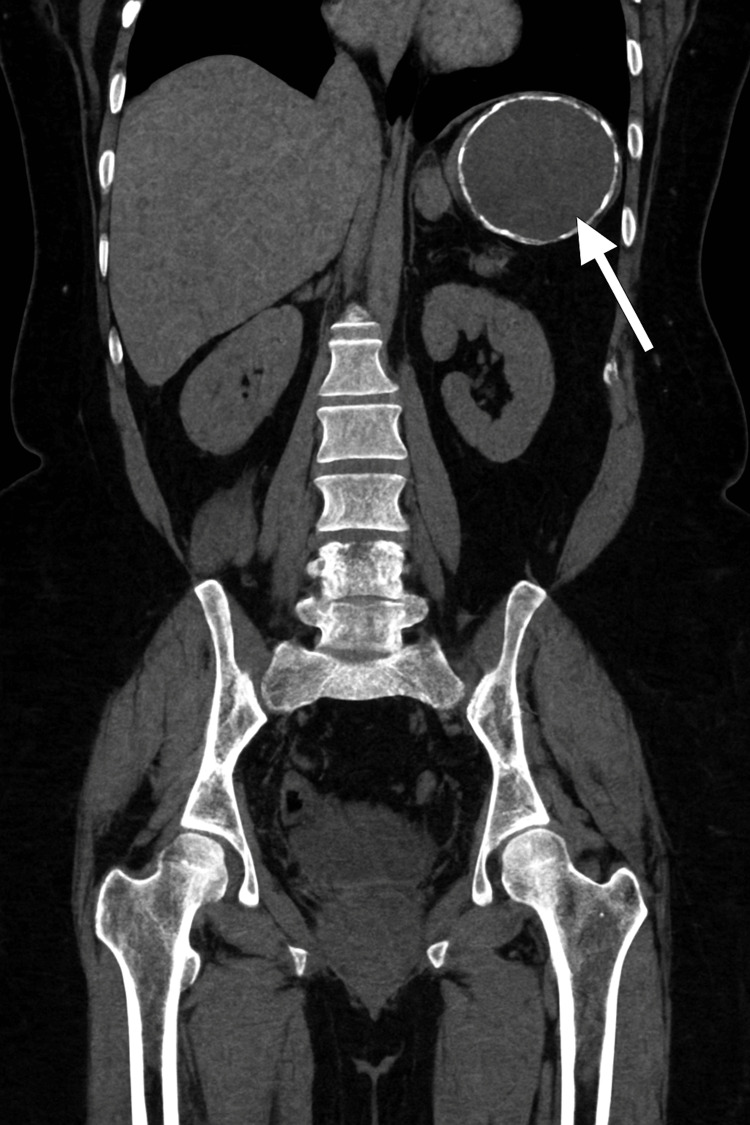
Coronal contrast-enhanced CT scan demonstrating a splenic epithelial cyst A well-defined, unilocular cystic lesion is seen in the upper pole of the spleen (white arrow), measuring approximately 6–7 cm, with no internal septations or solid components. Findings are consistent with a benign epithelial cyst.

A large, well-defined, unilocular splenic lesion is identified, measuring approximately 5.9 cm (AP) x 6.7 cm (TV) x 7.0 cm (CC). The lesion demonstrates fluid attenuation without internal solid components. In addition, peripheral discontinuous calcifications are present along the lesion wall (Figures 1, 2).

MRI findings

**Figure 3:**
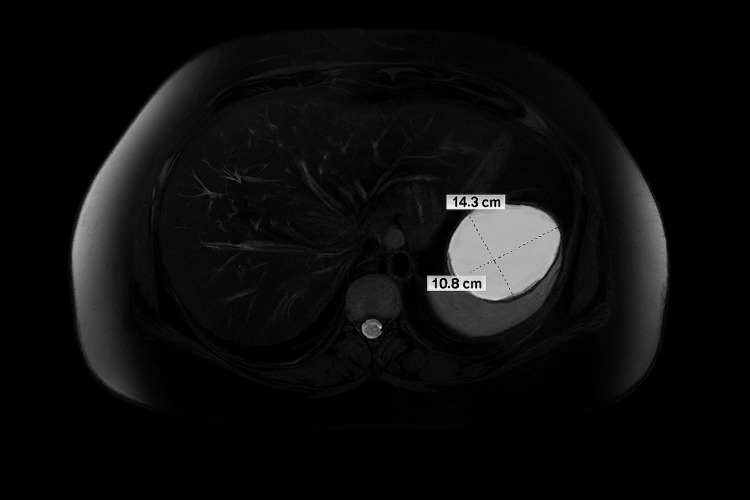
Axial T2-weighted MRI image of the upper abdomen showing a large, well-defined, unilocular exophytic splenic lesion measuring approximately 6.1 × 7.1 cm The lesion demonstrates high signal intensity with an internal fluid-fluid level and a thick wall of low T2 signal intensity, suggestive of a complex cystic lesion (possible splenic pseudocyst or epithelial cyst).

There is a corresponding large, unilocular, exophytic lesion in the spleen, measuring approximately 6.1 cm x 7.1 cm. On MRI, the lesion exhibits high signal intensity on both T2-weighted sequences, with a discernible fluid-fluid level. The wall of the lesion is thick and demonstrates low T2 signal intensity. These imaging characteristics suggest a complex cystic lesion, with a differential diagnosis that includes a splenic pseudocyst versus an epithelial cyst (Figure 3).

Postoperatively, CA 19-9 levels decreased significantly to 282.18 U/mL by day four and further dropped to 106.96 U/mL at the four-week follow-up. By the eighth postoperative week, the patient's CA 19-9 level had normalized,

## Discussion

Benign splenic epithelial cysts are true cysts that contain a cellular epithelial lining, most commonly mesothelial or squamous in origin. While rare, they represent the most frequent type of non-parasitic true splenic cysts [1-3]. Most cases are asymptomatic, but some present with compressive symptoms or become complicated by infection, hemorrhage, or rupture.

What distinguishes this case is the clear documentation of serial CA 19-9 levels, showing progressive normalization over an eight-week period following splenectomy. In contrast to previously reported cases [4,5], we provide a precise timeline of biochemical resolution, which reinforces the benign nature of the lesion and supports the hypothesis that CA 19-9 elevation in such cysts is directly related to cyst wall secretion [6,7]. Furthermore, the absence of hepatobiliary or pancreatic abnormalities alongside normal liver enzymes isolates the splenic cyst as the sole source of tumor marker elevation, strengthening the specificity of this association [3].

Suzuki et al. explored the relationship between intracystic pressure and epithelial integrity in relation to serum CA 19-9 levels, confirming that benign mechanisms can lead to falsely alarming tumor marker elevations [7]. Similarly, Kim et al. emphasized the need for a systematic approach to interpreting CA 19-9, highlighting benign causes including splenic and hepatic cysts in patients without malignancy [6].

In our case, the markedly elevated CA 19-9 level (627 U/mL) declined significantly after laparoscopic splenectomy, supporting the hypothesis of epithelial origin. This pattern has been consistently observed in similar reports, including those by Imoto et al. and Yoh et al., who described cases with normalization of tumor markers post-cyst resection [4,5].

Surgical resection remains the treatment of choice for symptomatic, large, or diagnostically ambiguous splenic cysts. Laparoscopic splenectomy offers a minimally invasive, definitive approach, allowing both symptom resolution and histological confirmation of benign pathology [3,5].

This case adds to the growing body of evidence that elevated CA 19-9 may occur in the context of benign splenic lesions and underlines the importance of correlating laboratory, radiologic, and histopathologic data to guide management appropriately.

## Conclusions

This case highlights the importance of a thorough diagnostic approach when confronted with elevated tumor markers such as CA 19-9, even in the absence of clinical or imaging signs of malignancy. Advanced cross-sectional imaging and histopathological confirmation are crucial in differentiating benign from malignant causes. In our patient, the normalization of CA 19-9 levels following laparoscopic splenectomy confirmed the benign nature of the lesion. Clinicians should consider splenic epithelial cysts in the differential diagnosis of isolated CA 19-9 elevation to avoid misdiagnosis and prevent unnecessary invasive procedures.
